# Comprehensive characterization of pyroptosis phenotypes with distinct tumor immune profiles in gastric cancer to aid immunotherapy

**DOI:** 10.18632/aging.204958

**Published:** 2023-08-17

**Authors:** Kaida Huang, Yubiao Lin, Guoqin Qiu, Shengyu Wang, Lihua Feng, Zhigao Zheng, Yingqin Gao, Xin Fan, Wenhui Zheng, Jianmin Zhuang, Fanghong Luo, Shuitu Feng

**Affiliations:** 1 Department of Oncology, Xiamen Haicang Hospital, Xiamen 361026, Fujian, China; 2 Chenggong Hospital Affiliated to Xiamen University, Xiamen 361003, Fujian, China; 3 Cancer Research Center, Medical College, Xiamen University, Xiamen 361102, China; 4 Department of General Surgery, Xiamen Haicang Hospital, Xiamen 361026, Fujian, China; 5 Fudan University Shanghai Cancer Center Xiamen Hospital, Xiamen 361000, Fujian, China

**Keywords:** gastric cancer, pyroptosis, immune response, tumor microenvironment, immunotherapy, prognosis

## Abstract

Objective: Pyroptosis is a form of programmed cell death that is essential for immunity. Herein, this study was conducted to uncover the implication of pyroptosis in immunomodulation and tumor microenvironment (TME) in gastric cancer.

Methods: Prognostic pyroptosis-related genes were extracted to identify different pyroptosis phenotypes and pyroptosis genomic phenotypes via unsupervised clustering analysis in the gastric cancer meta-cohort cohort (GSE15459, GSE62254, GSE84437, GSE26253 and TCGA-STAD). The activation of hallmark gene sets was quantified by GSVA and immune cell infiltration was estimated via ssGSEA and CIBERSORT. Through PCA algorithm, pyroptosis score was conducted. The predictors of immune response (TMB and IPS) and genetic mutations were evaluated. The efficacy of pyroptosis score in predicting immune response was verified in two anti-PD-1 therapy cohorts.

Results: Three different pyroptosis phenotypes with different prognosis, biological pathways and tumor immune microenvironment were established among 1275 gastric cancer patients, corresponding to three immune phenotypes: immune-inflamed, immune-desert, and immune-excluded. According to the pyroptosis score, patients were separated into high and low pyroptosis score groups. Low pyroptosis score indicated favorable survival outcomes, enhanced immune responses, and increased mutation frequency. Moreover, low pyroptosis score patients displayed more clinical benefits from anti-PD-1 and prolonged survival time.

Conclusion: Our findings uncovered a nonnegligible role of pyroptosis in immunomodulation and TME multiformity and complicacy in gastric cancer. Quantifying the pyroptosis score in individual tumors may tailor more effective immunotherapeutic strategies.

## INTRODUCTION

Gastric cancer ranks the third leading cause of deaths and the fifth most common malignancy globally, which remains a considerable health burden [1]. This disease is a molecularly heterogeneous entity [2]. Highly complex molecular features such as gene mutations and differential gene expression contribute to unfavorable survival outcomes of gastric cancer [3]. Common therapeutic strategies contain chemotherapy, radiotherapy, surgery resection as well as targeted therapies [4]. Immunotherapy has emerged as a new treatment strategy to eradicate malignant tumor cells and has received much attention after its successful clinical application [5]. Immune checkpoint inhibitors such as PD-1/PD-L1 monoclonal antibodies have prominently prolonged survival time of advanced gastric cancer [6–8]. Nevertheless, immunotherapy for gastric cancer is still in the early phases. Hence, it is of importance to clarify the immune regulation mechanisms, thus tailoring more effective immunotherapeutic scheme for individual patients.

Stromal and immune cells in the tumor microenvironment (TME) constitute an important part of tumor tissues [9]. Evidence has elucidated their clinicopathologic implication in prediction of prognosis and therapeutic response [10]. Recently, the novel concept of “immune phenotypes” has been proposed, stratifying tumors into three main immune profiles (inflamed, excluded, and deserted) with diverse TME traits and therapy options [11]. Hence, comprehensive analysis of the TME’s heterogeneity and complexity as well as immune traits may guide and predict immunotherapeutic response [12, 13]. Pyroptosis, a programmed cell death modality, can be caused by perturbation of extra- or intracellular homeostasis associated with innate immunity [14]. In morphology, pyroptosis exhibits the characteristics of cell swelling, lysis as well as the release of various proinflammatory mediators, e.g., IL-1β/-18 [15]. At the molecular level, pyroptosis primarily contains the classical pathway dependent on caspase-1 and the non-classical pathway involving caspase-4, 5 [16]. It has been confirmed that the targets and products of pyroptosis are involved in carcinogenesis, especially GSDM family members (GSDMs) [17]. Transforming immune “cold” tumors into “hot” tumors that more possibly respond to immunotherapy is the main strategy for cancer therapy [18]. Studies have found that pyroptosis may change the tumor immune microenvironment, ultimately enhancing response to immunotherapy [19]. Here, our study aimed at uncovering the implication of pyroptosis in immune modulation and diverse TME of gastric cancer, thus assisting therapeutic intervention plans.

## RESULTS

### Genetic and transcriptomic alterations of pyroptosis-related genes across gastric tumors

**Figure 1:**
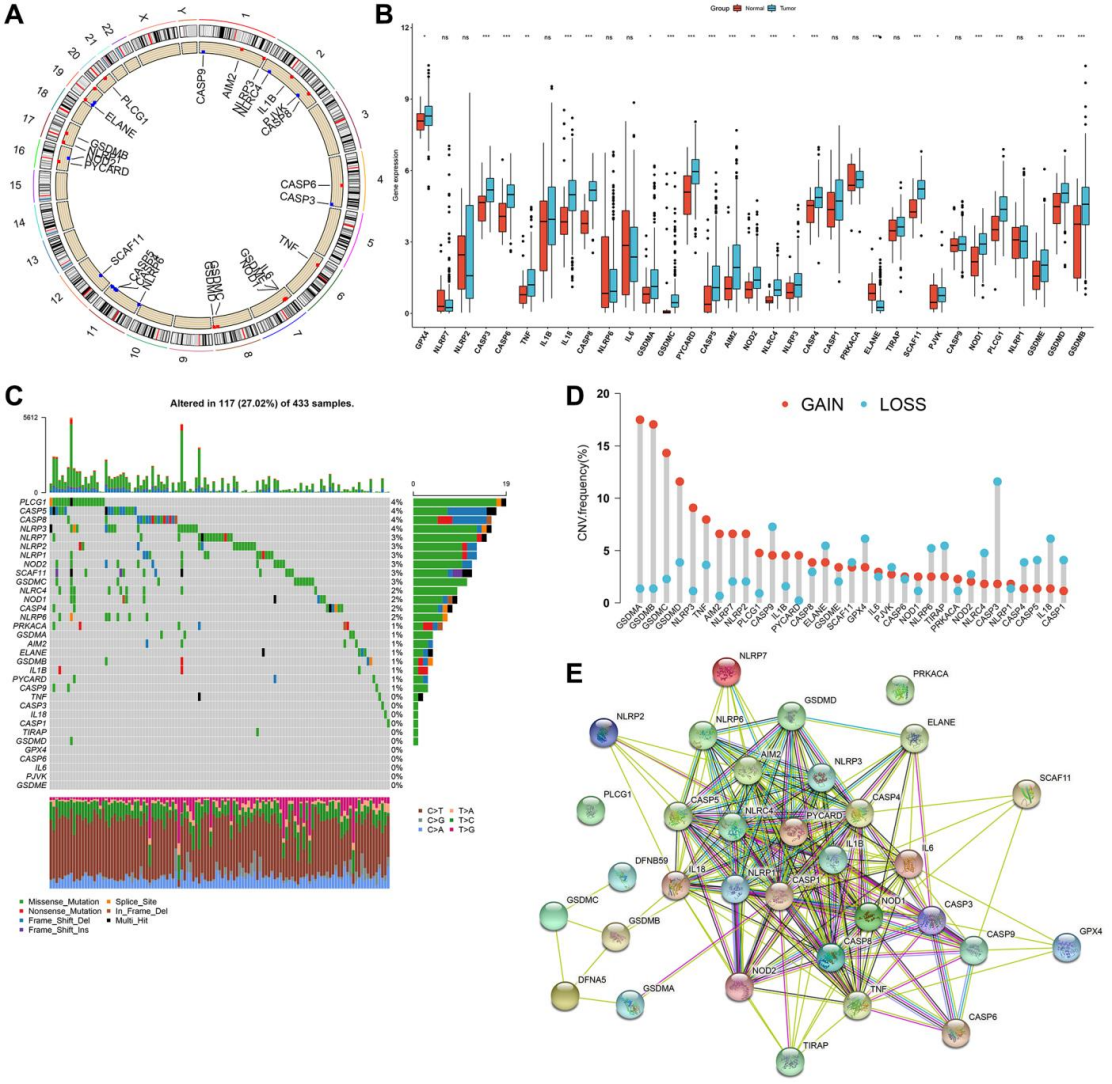
**Comprehensive characterization of genetic and expression alterations of pyroptosis-related genes in gastric cancer.** (**A**) Circos 2D track plots showing the location of 33 pyroptosis-related genes on chromosomes. (**B**) The mRNA expression of 33 pyroptosis-relevant genes in 32 normal and 375 gastric cancer tissue specimens from TCGA-STAD cohort. The asterisks indicated the statistical *p*-values (^*^p < 0.05; ^**^p < 0.01; ^***^p < 0.001) and ns indicated no statistical significance. (**C**) The somatic mutation frequency of 33 pyroptosis-relevant genes in 433 gastric cancer samples in TCGA-STAD dataset. The upper barplot displayed somatic mutation. The stacked barplot exhibited the fractions of conversions of specimens. The number on the right showed the mutation frequency in each pyroptosis-related gene. The right barplot showed the proportion of each mutation type. (**D**) Summary of the CNV frequency of 33 pyroptosis-related genes. The column height indicated the mutation frequency. Amplification, red; deletion, blue. (**E**) The PPI network visualizing the interactions between 33 pyroptosis-relevant genes.

Here, we observed the functions of 33 pyroptosis-relevant genes in gastric tumors. We firstly identified their chromosomal locations (Figure 1A). The mRNA expression levels of above genes were investigated in 32 controls and 375 gastric tumors from TCGA-STAD cohort. The results showed that most of pyroptosis-related genes had higher expression levels in tumors (Figure 1B). The incidence of somatic mutations of pyroptosis-related genes in gastric cancer was then summarized. Among 433 specimens, 117 occurred somatic mutations of pyroptosis-associated signatures, with frequency of 27.02% (Figure 1C). We investigated that PLCG1, CASP5, CASP8 and NLRP3 displayed the highest mutation frequencies in gastric cancer specimens. Further analysis exhibited the widespread CNV frequencies in 33 pyroptosis-related genes and most were focused on the amplification in CNV (Figure 1D). These findings indicated that such genetic variations affected their instability in gastric tumors. The PPI network showed the extensive interplay of such pyroptosis-relevant genes (Figure 1E), indicative of the potential implication of their cross-talk in gastric cancer pathogenesis and progression.

### Identification of three distinct pyroptosis phenotypes in gastric cancer

**Figure 2:**
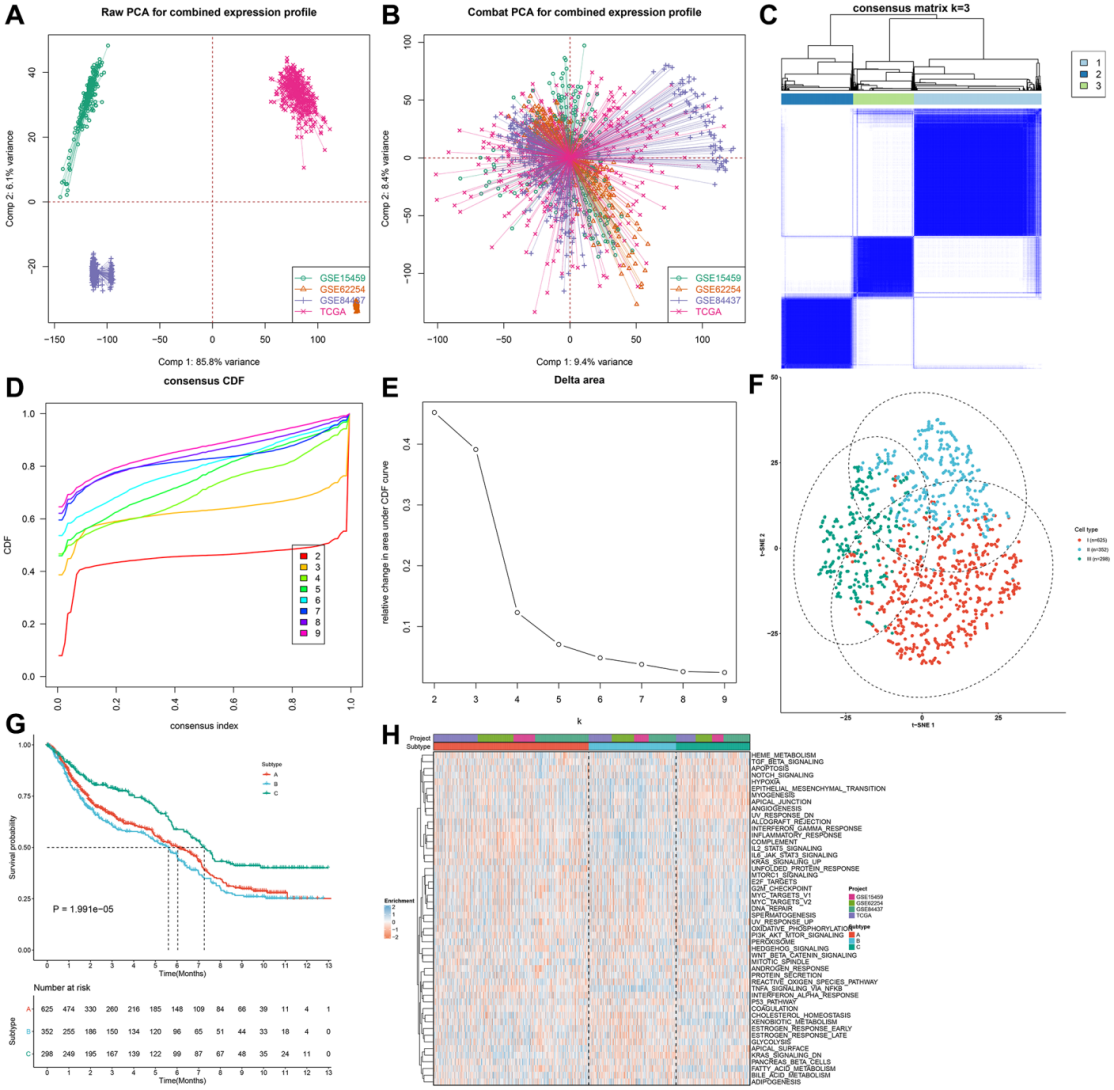
**Identification of three distinct pyroptosis phenotypes in gastric cancer.** (**A**, **B**) Integration of four gastric cancer cohorts (GSE15459, GSE62254, GSE84437 and TCGA-STAD) into one meta-cohort and removal of batch effects. (**C**) Heat map showing the optimal clustering by k = 3 based on prognostic pyroptosis-related genes in the meta-cohort. (**D**) The consensus cumulative distribution function (CDF) for the cumulative distributive function of the consensus matrix for k = 2–9 identified by different colors. (**E**) The CDF plots of consensus clustering matrix for k = 2–9. (**F**) The t-SNE plots of the mRNA expression profiles of prognostic pyroptosis-related genes showing the three distinct subtypes indicated by different colors. (**G**) Kaplan–Meier survival curves for the three pyroptosis subtypes based on the 1275 gastric cancer specimens in the meta-cohort (log-rank test). (**H**) Heatmap showing the GSVA score of the 50 hallmark pathways in three pyroptosis phenotypes.

Four gastric cancer cohorts (GSE15459, GSE62254, GSE84437 and TCGA-STAD) were integrated and batch effects were removed (Figure 2A, 2B). Finally, 1275 gastric cancer patients were integrated as the meta-cohort. Univariate-cox regression analysis of 33 pyroptosis-related genes showed that GPX4 (HR: 1.24 (1.06–1.46); *p* = 0.008), NLRP7 (HR: 0.86 (0.76–0.96; *p* = 0.009), CASP6 (HR: 0.87: (0.76–0.99); *p* = 0.032), NLRP6 (HR: 0.90 (0.82–0.99); *p* = 0.04), IL6 (HR: 1.07 (1.00–1.13); *p* = 0.047), CASP5 (HR: 0.90 (0.82–0.99); *p* = 0.023), CASP4 (HR: 0.84 (0.71–0.999); *p* = 0.048) and TIRAP (HR: 0.73 (0.59–0.91); *p* = 0.004) had significant correlations to prognosis across 1275 gastric cancer patients. Through the ConsensusClusterPlus package, we stratified gastric cancer specimens to three different pyroptosis phenotypes according to the expressions of above 8 prognostic pyroptosis-related genes with unsupervised clustering method (Figure 2C–2E; Supplementary Table 1). We named these phenotypes as pyroptosis cluster A (*n* = 625), B (*n* = 352) and C (*n* = 298). There were remarkable differences in the transcriptional profiles of the prognostic pyroptosis-relevant gene set among three diverse pyroptosis phenotypes (Figure 2F). Prognostic analysis for the three pyroptosis phenotypes showed the particularly prominent survival advantage in pyroptosis cluster-C, while pyroptosis cluster-B displayed the poorest survival outcomes (Figure 2G). The accuracy of this pyroptosis-related classification was externally proven in the GSE26253 (Supplementary Figure 1A–1D). The differences in prognosis among three pyroptosis phenotypes were also confirmed in the GSE26253 cohort (Supplementary Figure 1E).

### Pyroptosis phenotypes with different immune profiles

**Figure 3:**
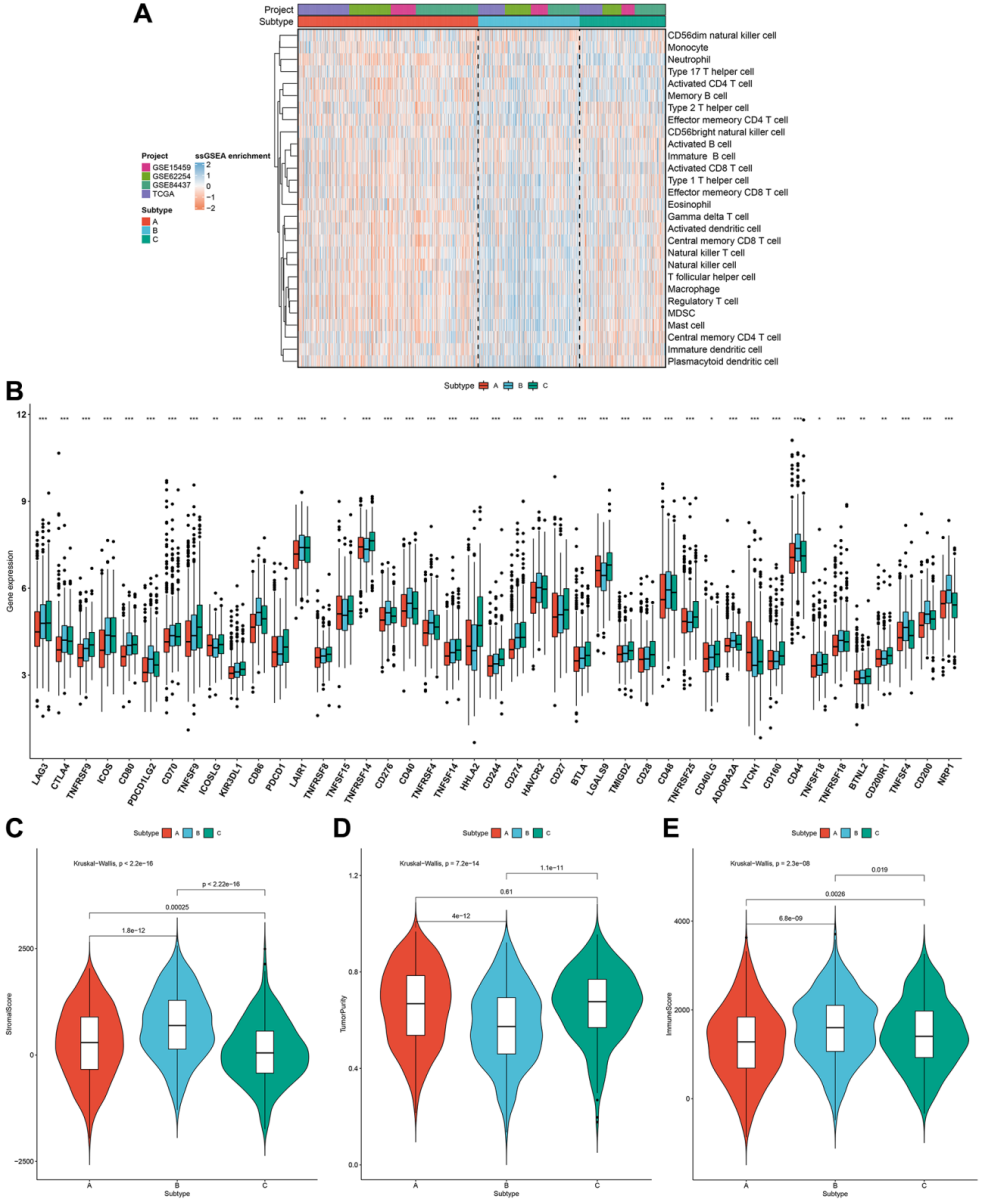
**Pyroptosis phenotypes with different immune landscapes in the gastric cancer meta-cohort.** (**A**) The relative abundance of 28 immune cells in the TME among distinct pyroptosis phenotypes through ssGSEA. (**B**) The mRNA expression of the main immune checkpoints among three pyroptosis phenotypes. The asterisks indicated the statistical *p*-values (^*^p < 0.05; ^**^p < 0.01; ^***^p < 0.001). (**C**–**E**) The stromal score, tumor purity and immune score in three pyroptosis phenotypes.

For uncovering potential biomolecular alterations in three different pyroptosis phenotypes, this study carried out GSVA on these 50 hallmark gene sets. We investigated that pyroptosis cluster-C owned distinct enrichment by stromal pathways (e.g., TGF-β signaling, epithelial mesenchymal transition and angiogenesis; Figure 2H). Pyroptosis cluster-B was highly enriched in carcinogenic activation pathways such as PI3K-AKT-mTOR signaling, Wnt-β-catenin signaling, glycolysis and KRAS signaling. Moreover, pyroptosis cluster-A markedly displayed the activation of immune-relevant processes like allograft rejection, interferon gamma response, inflammatory response, complement, IL2-STAT5 signaling and IL6-JAK-STAT3 signaling. These data were indicative of the survival outcomes of the three pyroptosis phenotypes. Moreover, ssGSEA was adopted for estimating the abundant levels of 28 immune cell populations within the TME among distinct pyroptosis phenotypes. Immune-excluded phenotype is rich in immune cells that are reserved in the matrix ambient the tumor cell nest instead of penetrating its parenchyma [20]. Pyroptosis cluster-C could be categorized as immune-excluded tumors with innate immune cell infiltrations as well as stromal activation (Figure 3A). The cluster-B could be categorized as immune-desert tumors with low infiltration levels of nearly all immune cells, indicative of immunity suppression. Moreover, the cluster-A could be categorized as immune-inflamed tumors with rich adaptive immune cells as well as immune activation. The immune infiltration traits across three pyroptosis phenotypes were confirmed via CIBERSORT algorithm (Supplementary Figure 2). Figure 3B showed the prominent heterogeneity in immune checkpoints among pyroptosis phenotypes. Pyroptosis cluster-C exhibited the highest expression of LAG3, CTLA4, TNFRSF9, ICOS, CD80, TNFSF9, ICOSLG, KIR3DL1, PDCD-1, TNFRSF8, TNFRSF15, TNFRSF14, HHLA2, CD244, CD27, BTLA, LGALS9, TMIGD2, CD28, TNFRSF25, CD40LG, CD160, and CD200R1. Through ESTIMATE algorithm, we quantified the entire infiltrations of immune/stromal cells or tumor purity among the pyroptosis phenotypes. Our results showed that pyroptosis cluster-B had the highest stromal score, followed by pyroptosis cluster-A and pyroptosis cluster-C (Figure 3C). Inversely, pyroptosis cluster-B displayed the lower tumor purity, indicating that tumors in the cluster-B were potentially neighbored by rich non-cancer cells such as stromal/immune cells (Figure 3D). In Figure 3E, pyroptosis cluster-B had the highest immune score, followed by pyroptosis cluster-A and pyroptosis cluster-C. Collectively, our data demonstrated that the different pyroptosis phenotypes had the features of distinct immune landscapes.

### Identification of pyroptosis phenotype-related gene signatures

**Figure 4:**
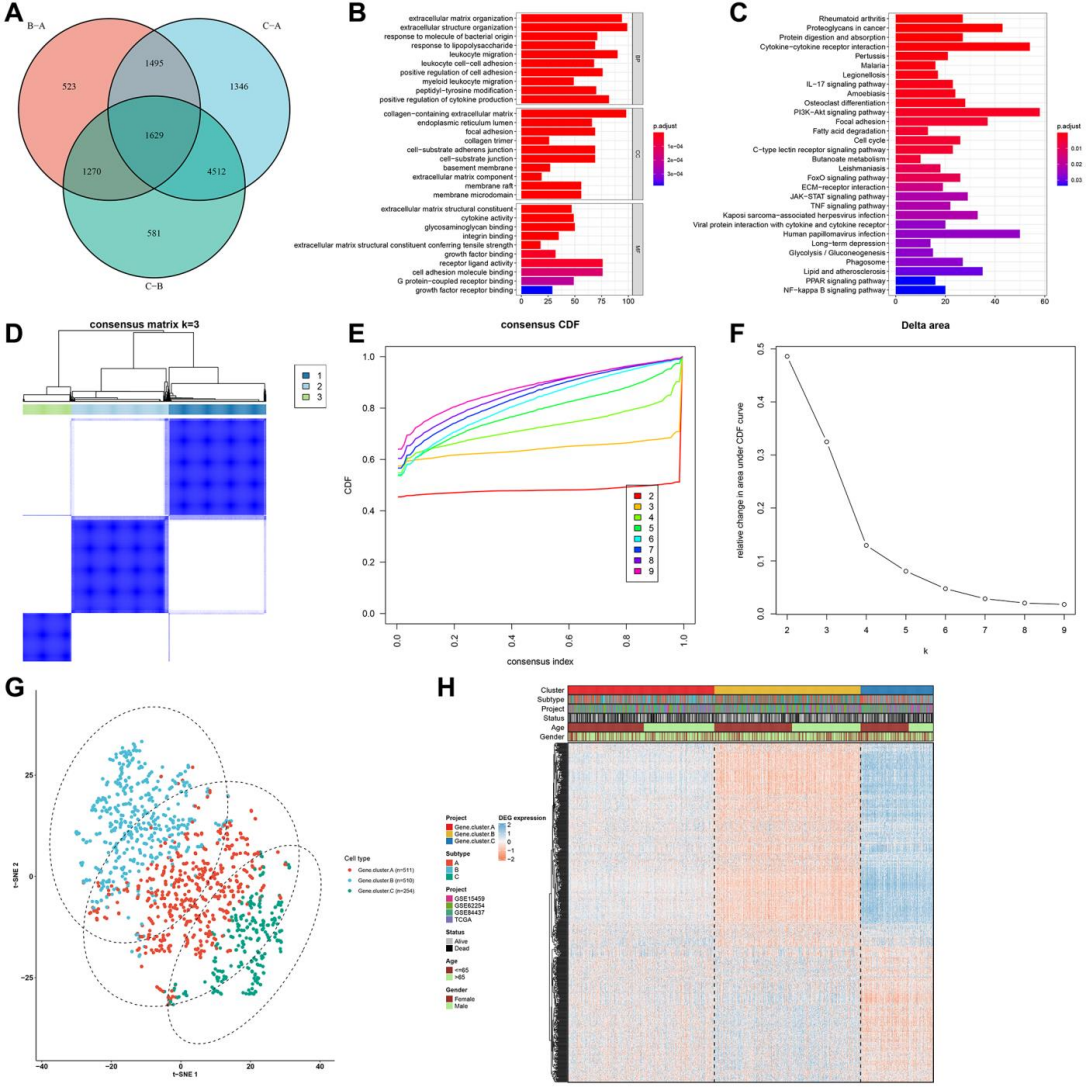
**Identification of pyroptosis phenotype-related gene signatures in the gastric cancer meta-cohort.** (**A**) Venn diagram showing the overlapped pyroptosis phenotype-related DEGs among three pyroptosis phenotypes. (**B**, **C**) GO and KEGG enrichment results of the overlapped pyroptosis phenotype-related DEGs. (**D**) Heat map showing the optimal clustering by k = 3 based on the overlapped pyroptosis phenotype-related DEGs. (**E**) The CDF for the cumulative distributive function of the consensus matrix for k = 2–9 identified by different colors. (**F**) The CDF plots of consensus clustering matrix for k = 2–9. (**G**) The t-SNE plots of the mRNA expression of pyroptosis phenotype-related gene signatures visualizing three distinct pyroptosis genomic phenotypes. (**H**) Heatmap showing the differences in the expression of pyroptosis phenotype-related gene signatures and clinicopathological characteristics among three pyroptosis genomic phenotypes.

The pyroptosis phenotype-related transcriptional expression alterations across three pyroptosis phenotypes were detected in the gastric cancer meta-cohort. Through limma package, we identified 1629 overlapped pyroptosis phenotype-relevant DEGs among the three phenotypes (Figure 4A; Supplementary Table 2). These DEGs represented the key distinguishing indicators for three pyroptosis phenotypes. GO enrichment analyses of above DEGs suggested that immunity-relevant mechanisms presented the marked enrichment (Figure 4B, 4C). This indicated that such DEGs were pyroptosis phenotype-relevant genes. According to the 1629 pyroptosis phenotype-relevant gene signatures, this study established three reliable genomic phenotypes (Figure 4D–4G). Gastric cancer patients were clustered into three different pyroptosis genomic phenotypes, named as pyroptosis gene cluster-A (*n* = 511), cluster-B (*n* = 510) and cluster-C (*n* = 254). Moreover, Figure 4H shows the prominent heterogeneity in pyroptosis phenotype-associated genes and clinicopathological characteristics among three pyroptosis genomic phenotypes.

### Pyroptosis genomic phenotypes with distinct prognosis and immune landscapes

**Figure 5:**
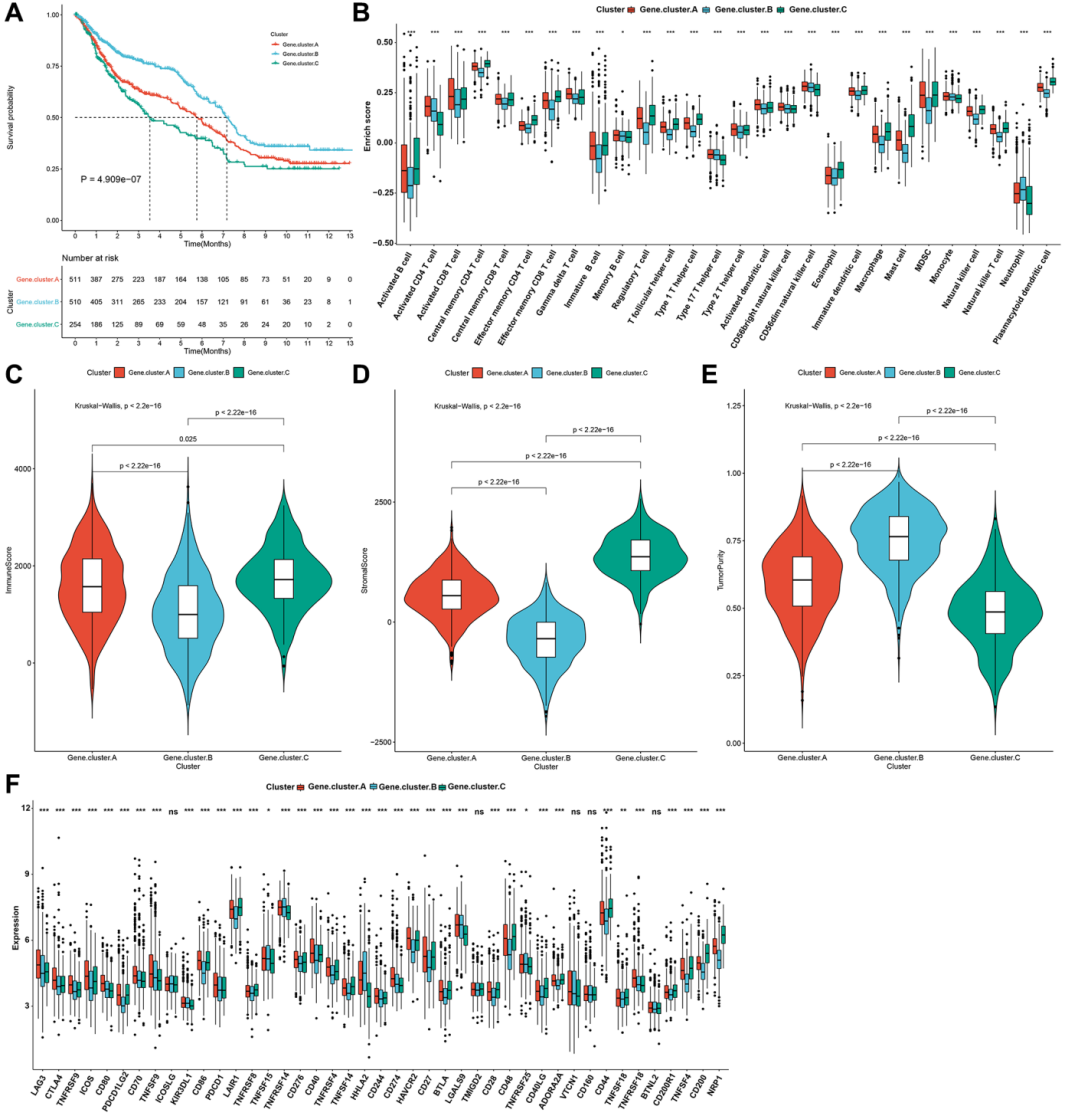
**Pyroptosis genomic phenotypes with distinct prognosis and immune landscapes in the gastric cancer meta-cohort.** (**A**) Kaplan-Meier survival curves for three pyroptosis genomic phenotypes (log-rank test). (**B**) The relative abundance of 28 immune cells among three pyroptosis genomic phenotypes through ssGSEA. (**C**–**E**) The immune score, stromal score, and tumor purity across three genomic phenotypes. (**F**) The mRNA expression of immune checkpoints across three genomic phenotypes. The asterisks indicated the statistical *p*-values (^*^p < 0.05; ^***^p < 0.001) and ns indicated not significant.

Survival analysis showed the prominent prognostic differences among three pyroptosis genomic phenotypes in the gastric cancer meta-cohort. Pyroptosis gene cluster-C owned the poorest prognosis, while pyroptosis gene cluster-B exhibited a significant survival advantage (Figure 5A). Consistent with pyroptosis phenotypes, pyroptosis genomic cluster-C could be classified into immune-excluded tumors with innate immune cell infiltrations as well as stromal cells, with the genomic cluster-B categorized into immune-desert tumors with immunity suppression, and the genomic cluster-A categorized into immune-inflamed tumors with rich adaptive immune cells as well as immune activation (Figure 5B–5E). The heterogeneity in transcriptomic levels of immune checkpoints was also investigated across three pyroptosis genomic phenotypes (Figure 5F).

### Generation of the pyroptosis score and evaluation of its clinical implication

**Figure 6:**
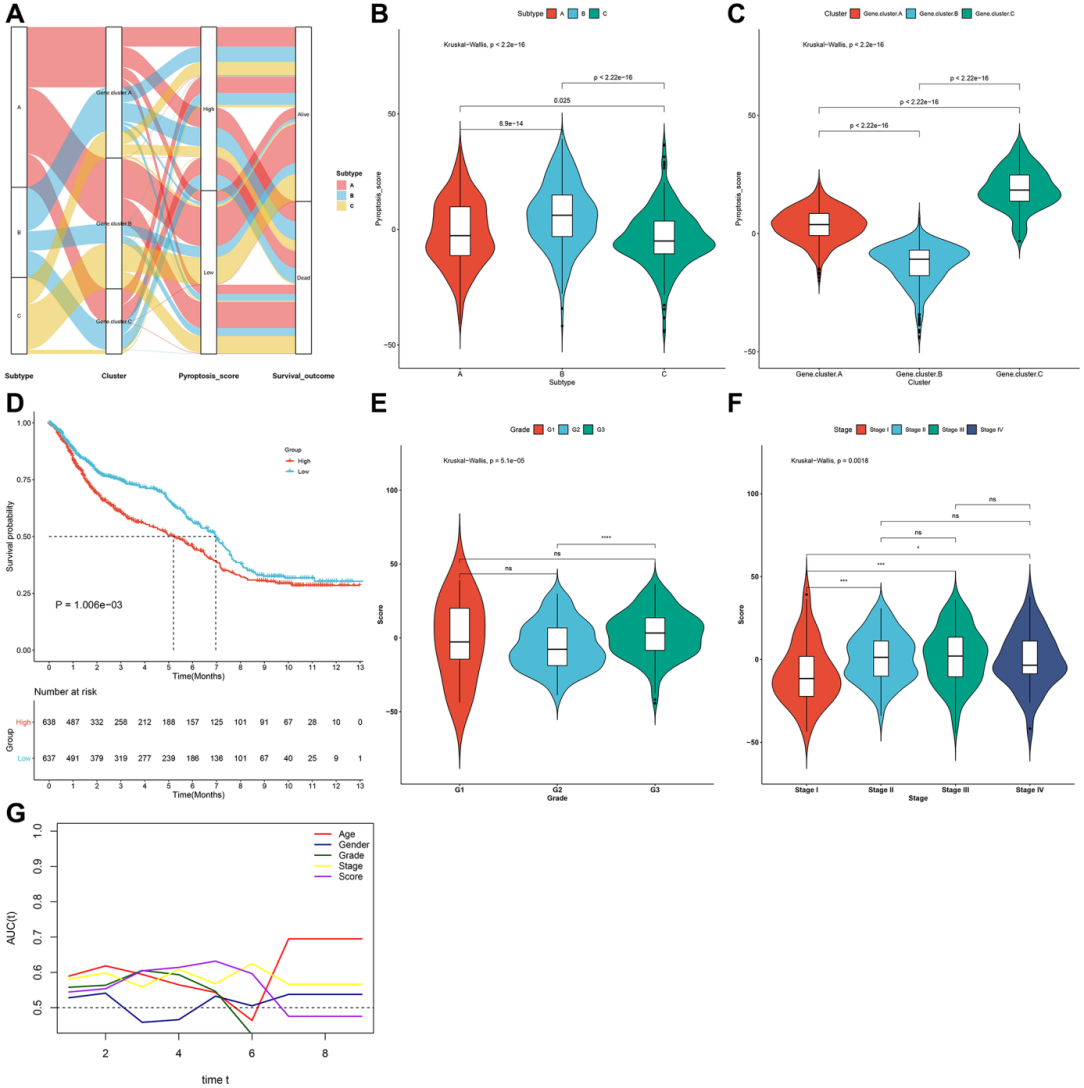
**Generation of the pyroptosis score and evaluation of its clinical implication in the gastric cancer meta-cohort.** (**A**) Alluvial diagram of pyroptosis phenotypes with different pyroptosis genomic phenotypes, pyroptosis score and survival status. (**B**) Distribution of pyroptosis score in three pyroptosis phenotypes. (**C**) Distribution of pyroptosis score in three pyroptosis genomic phenotypes. (**D**) Kaplan-Meier survival curves for high and low pyroptosis score groups (log-rank test). (**E**) Distribution of pyroptosis score in different grades (G1-3). (**F**) Distribution of pyroptosis score in different stages (stage I–IV). (**G**) Time-independent ROC curves showing the predictive performance of the pyroptosis score, age, gender, grade, and stage in gastric cancer prognosis.

To accurately quantify pyroptosis phenotypes in individual gastric cancer, this study proposed a scoring system named the pyroptosis score according to the pyroptosis phenotype-relevant gene signatures with PCA algorithm. Alluvial diagram showed the heterogeneity of the quantification of pyroptosis score among different pyroptosis phenotypes, pyroptosis genomic phenotypes and survival status (Figure 6A). Notably, pyroptosis cluster-B owned the strongest pyroptosis score, with subsequent cluster-A or cluster-C (Figure 6B). Moreover, pyroptosis genomic cluster-C displayed the strongest pyroptosis score, with subsequent cluster-A or cluster-B (Figure 6C). Next, gastric tumors in the meta-cohort were stratified as low or high pyroptosis score group. The efficiency of the scoring system in inferring survival outcomes was then observed. In Figure 6D, high pyroptosis score displayed poorer survival. The pyroptosis score distribution across different clinical characteristics was also analyzed. Patients with advanced grade and stage had higher pyroptosis score (Figure 6E, 6F). Further analysis of time-independent ROC curves confirmed the well performance of the scoring system (Figure 6G). Thus, the scoring system could be utilized for evaluating certain clinicopathological characteristics (grade and stage) of gastric cancer patients.

### Characteristics of the pyroptosis score in molecular subtypes and tumor genome somatic mutations

**Figure 7:**
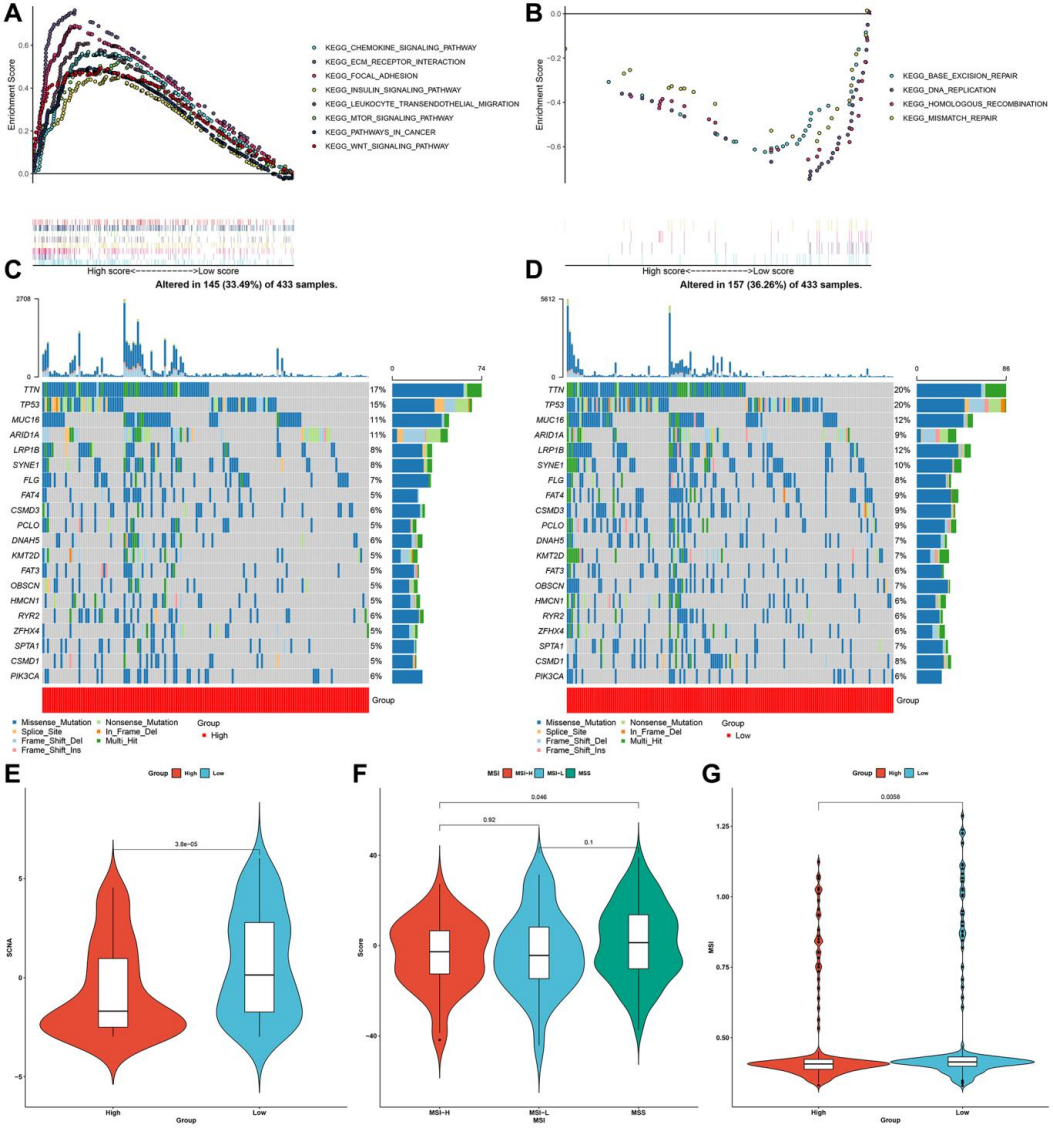
**Characteristics of the pyroptosis score in molecular subtypes and tumor genome somatic mutations.** (**A**, **B**) GSEA showing the activated signaling pathways in samples with high and low pyroptosis scores in the gastric cancer meta-cohort. (**C**, **D**) Mutational landscape in TCGA-STAD cohort clustered by high and low pyroptosis score subgroups. (**E**) Relative distribution of SCNA in high and low pyroptosis score groups from TCGA-STAD cohort. (**F**) Relative distribution of pyroptosis score in different molecular subtypes (MSI-H, MSI-L and MSS) in TCGA-STAD cohort. (**G**) Relative distribution of MSI status in patients with high and low pyroptosis scores from TCGA-STAD cohort.

GSEA results uncovered that high pyroptosis score was significantly connected to oncogenic or immune-related pathways (Figure 7A), whereas low pyroptosis score exhibited significant associations with genetic mutations (Figure 7B). Significantly mutated genes were compared in two groups. As shown in mutational landscape, higher somatic mutational frequency was found in low pyroptosis score group (33.49% vs. 36.26%; Figure 7C, 7D). TTN (17% vs. 20%), TP53 (15% vs. 20%), MUC16 (11% vs. 12%), etc. exhibited increased genetic mutation frequencies for patients with low pyroptosis score. Somatic copy number alterations (SCNA) exhibit positive correlation to immune evasion as well as proliferative capacity of tumor cells. We observed an increased SCNA for patients with low pyroptosis score (Figure 7E). The high microsatellite instability (MSI-H) subtype, characterized by favorable survival outcomes exhibited distinct correlation to lower pyroptosis score, while low MSI (MSI-L) and microsatellite stable (MSS) status displayed higher pyroptosis score (Figure 7F, 7G).

### The pyroptosis score predicts immunotherapeutic responses and chemosensitivity

**Figure 8:**
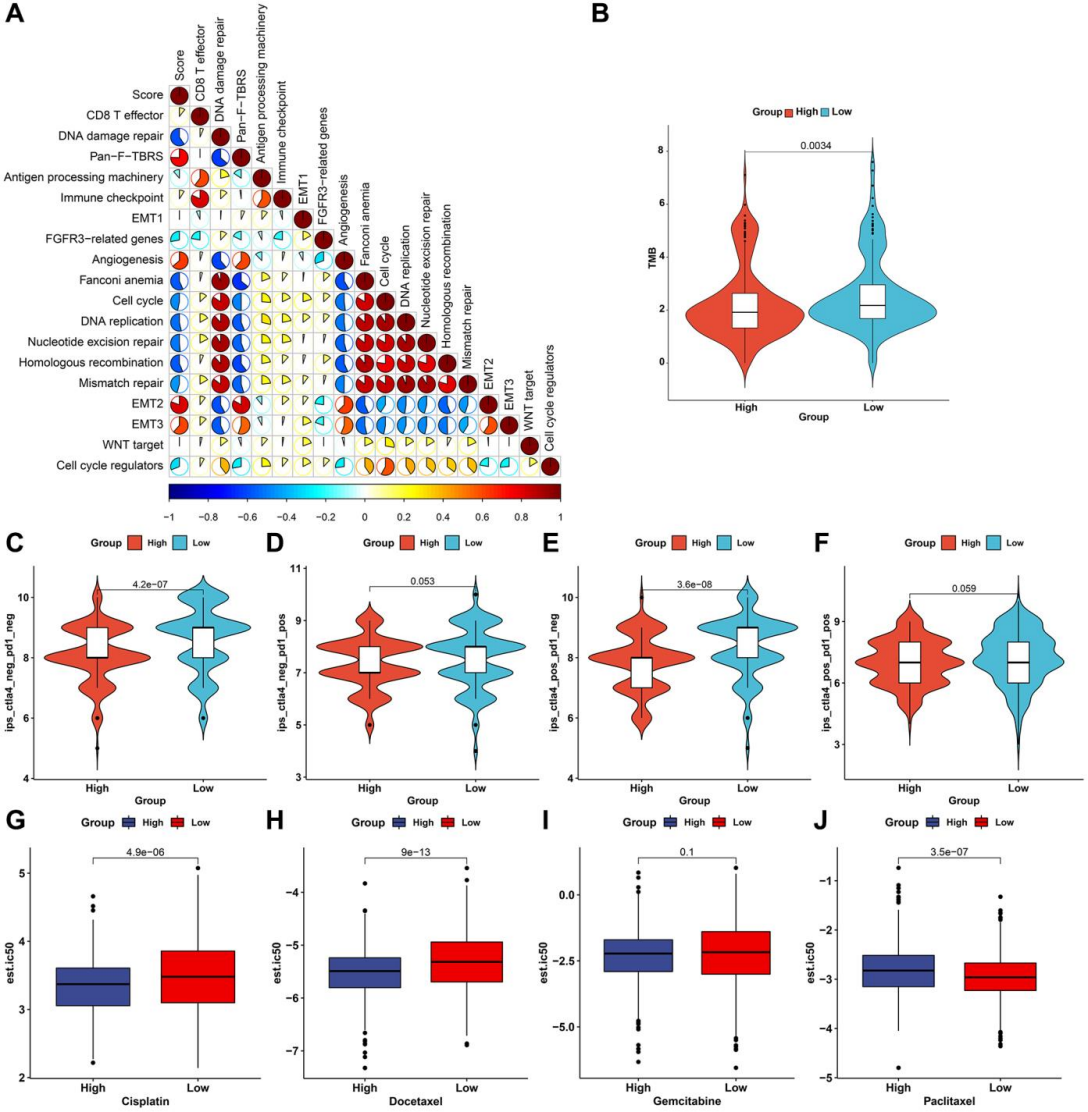
**The roles of the pyroptosis score in predicting immunotherapeutic responses and chemosensitivity.** (**A**) Interactions of the pyroptosis score with several signaling pathways through Spearman analyses. Red, positive correlation and blue, negative correlation. (**B**) Distribution of TMB score in high and low pyroptosis score groups in TCGA-STAD cohort. (**C**–**F**) Distribution of IPS score of CTLA-4/PD-1 in high and low pyroptosis score groups in TCGA-STAD cohort. (**G**–**J**) The estimated IC50- values of cisplatin, docetaxel, gemcitabine, and paclitaxel in patients with high and low pyroptosis scores in the meta-cohort.

**Figure 9:**
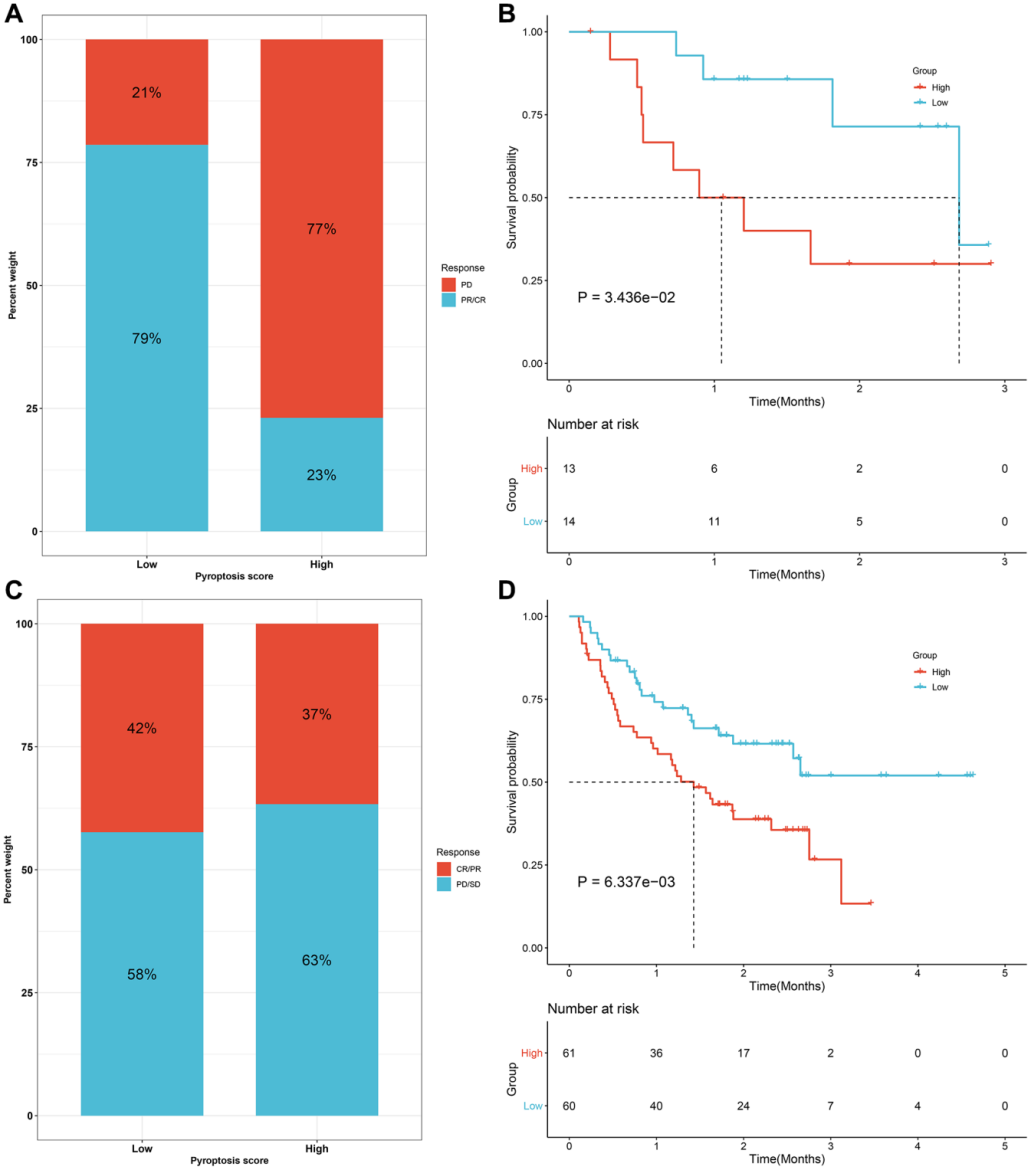
**Validation of the predictive efficacy of the pyroptosis score for immunotherapeutic responses in two anti-PD-1 cohorts.** (**A**) The fraction of patients with clinical response to anti-PD-1 therapy in high and low pyroptosis score groups from the GSE78220 cohort. (**B**) Kaplan-Meier curves for patients with high and low pyroptosis scores in the GSE78220 cohort (log-rank test). (**C**) The fractions of patients who presented therapeutic responses to anti-PD-1 therapy in high and low pyroptosis score groups from the Liu cohort. (**D**) Kaplan-Meier curves of patients with high and low pyroptosis scores in the Liu cohort (log-rank test). Abbreviations: CR: complete response; PR: partial response; SD: stable disease; PD: progressive disease.

We evaluated the interaction of the pyroptosis score with several signaling pathways by Spearman analyses. Consequently, the pyroptosis score presented negative correlations to DNA damage repair processes but was positively correlated to stromal-related signatures in the meta-cohort (Figure 8A). Immunotherapies like CTLA-4/PD-1 blockage have strictly made progress for anti-cancer treatment. Tumor mutation burden (TMB) has become an effective biomarker for predicting immunotherapeutic response. Low pyroptosis score presented notable connection to increased TMB in TCGA-STAD cohort (Figure 8B). IPS is also recommended as an estimator of immunotherapeutic efficacy. The analysis revealed that IPS score of CTLA-4/PD-1 inhibitor treatment was distinctly elevated in low pyroptosis score in TCGA-STAD cohort (Figure 8C–8F). The response to common chemotherapeutic agents was estimated in each sample from the meta-cohort (Figure 8G–8J). We observed that high pyroptosis score exhibited markedly reduced IC50 values of cisplatin and docetaxel as well as low pyroptosis score had significantly increased IC50 value of paclitaxel. Thus, patients with high pyroptosis score could be more sensitive to cisplatin and docetaxel as well as low pyroptosis score was more likely to benefit from paclitaxel. Due to the close association between the pyroptosis score and immune response, this study further observed the predictive performance of the pyroptosis score in immunotherapeutic responses in two anti-PD-1 cohorts. The prominent therapeutic benefits and immune responses to PD-1 inhibitor therapy were confirmed in low pyroptosis score group (79%) than another group (21%) in the GSE78220 cohort (Figure 9A). Patients with low pyroptosis score displayed prominent survival advantages (Figure 9B). The consistent results also confirmed that low pyroptosis score was distinctly correlated to clinical benefits and prolonged survival time in another anti-PD-1 therapy (Liu et al. study; Figure 9C, 9D). Collectively, above data prominently demonstrated that the pyroptosis score could estimate immunotherapeutic efficiency.

### Validating prognostic pyroptosis-relevant genes in gastric tumors

**Figure 10:**
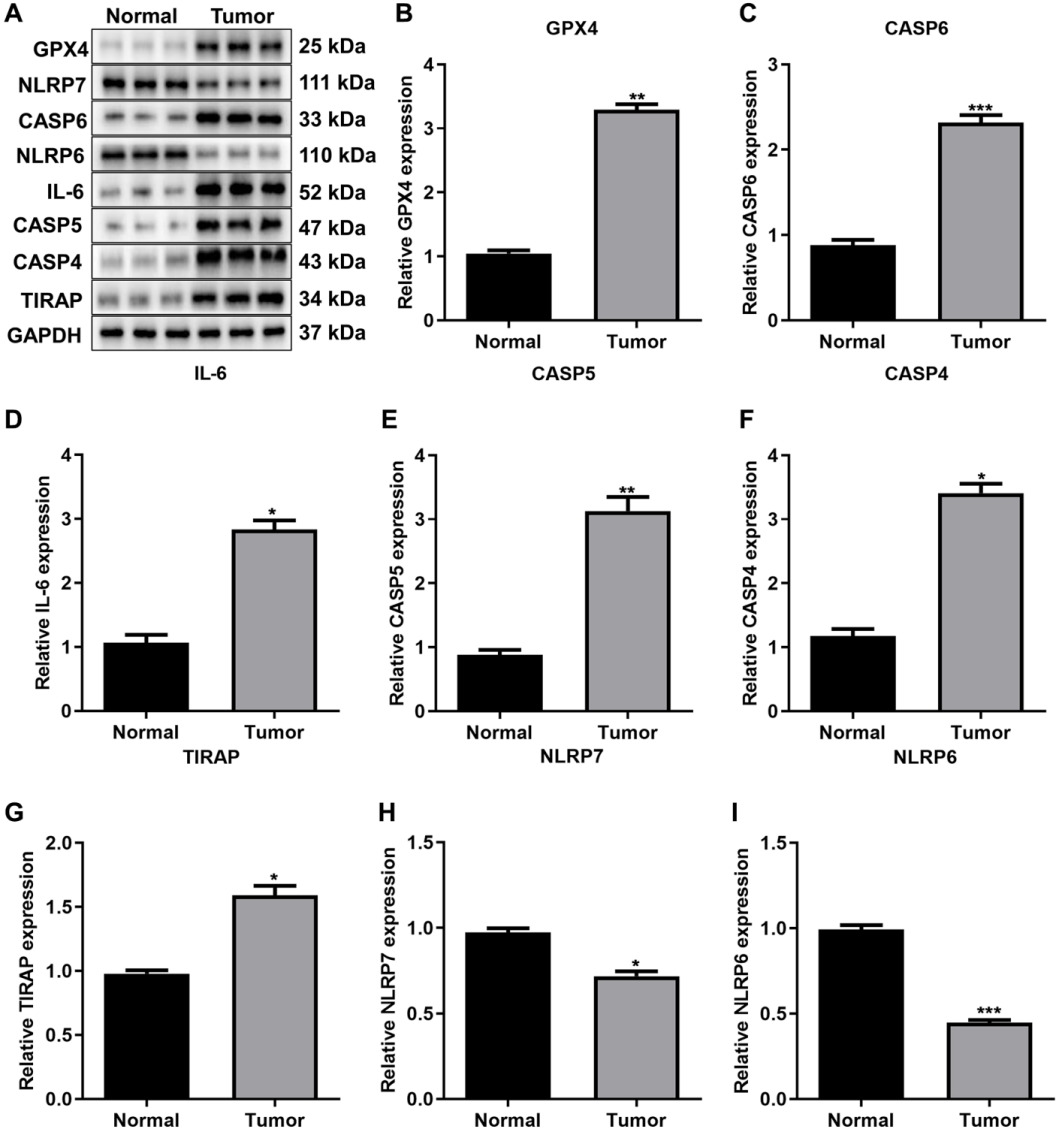
**Validation of the expression of prognostic pyroptosis-related genes.** (**A**–**I**) Western blot for the expression of (**B**) GPX4, (**C**) CASP6, (**D**) IL-6, (**E**) CASP5, (**F**) CASP4, (**G**) TIRAP, (**H**) NLRP7 and (**I**) NLRP6 in three paired gastric cancer tissues and normal tissues. The asterisks indicated the statistical *p*-values (^*^p < 0.05; ^**^p < 0.01; ^***^p < 0.001).

Through western blot, we validated the transcriptomic levels of such prognostic pyroptosis-relevant genes in three paired gastric cancer tissues and normal tissues (Figure 10A). Our results confirmed that the expression of GPX4 (Figure 10B), CASP6 (Figure 10C), IL-6 (Figure 10D), CASP5 (Figure 10E), CASP4 (Figure 10F), and TIRAP (Figure 10G) was greatly up-regulated in gastric tumors relative to normal tissues. Meanwhile, the expression of NLRP7 (Figure 10H) and NLRP6 (Figure 10I) was greatly attenuated in gastric cancer tissues than controls. Above evidence confirmed the abnormal expression of the prognostic pyroptosis-related genes in gastric cancer.

## DISCUSSION

Pyroptosis is a programmed cell death form, triggered by perturbation of extra- or intracellular homeostasis linked to innate immunity [21]. Transforming immune “cold” tumors into “hot” tumors is a main strategy to improve the responses to immunotherapy. Accumulated evidence has demonstrated that pyroptosis may change the tumor immune microenvironment, ultimately enhancing response to immunotherapy [19]. Although many findings have suggested the modulation of pyroptosis in tumoral immunity, the overall TME traits regulated by pyroptosis don’t remain fully understood. Hence, identification of different pyroptosis phenotypes in the tumor immune microenvironment may offer an insight into the roles of pyroptosis on anti-cancer immune responses as well as boost more favorable immunotherapeutic schemes.

Herein, our research established three different pyroptosis phenotypes with diverse survival outcomes and immune phenotypes. Pyroptosis cluster-C owned the prominent survival advantage, with subsequent pyroptosis cluster-A as well as cluster-B. The cluster-A presented the features of adaptive immune cell infiltrations and immune activation, which corresponded to an immune-inflamed tumor; the cluster-B had the characteristics of immunity suppression, which corresponded to an immune-desert phenotype; the cluster-C exhibited innate immune cell infiltrations as well as stromal activation, which corresponded to an immune-excluded phenotype. This work showed that pyroptosis cluster-C possessed correlations to stromal pathway activation (like TGF-β signaling, epithelial mesenchymal transition and angiogenesis) [22]. It has been suggested that the activation of TGF-β- and epithelial mesenchymal transition-related pathways prevents lymphocytes from penetrating the tumor parenchyma [23]. Targeting TGF-β can reshape the TME and enhance the anti-tumor immunity [24]. Thus, we inferred that gastric cancer patients in pyroptosis cluster-C could benefit from the combination of immunotherapy and TGF-β inhibitors. Pyroptosis cluster-A presented the activation of immune-related processes. Pyroptosis cluster-B was significantly correlated to carcinogenic activation pathways such as PI3K-AKT-mTOR signaling, Wnt-β-catenin signaling, glycolysis and KRAS signaling, indicating poor prognosis of patients in pyroptosis cluster-B.

Totally, we identified 1629 overlapped pyroptosis phenotype-relevant genes across pyroptosis phenotypes, which were significantly associated with immunity-related biological processes and pathways, demonstrating that above genes were pyroptosis phenotype-relevant genes. Consistently, we constructed three different pyroptosis genomic phenotypes characterized by different prognosis and immune landscape. Although this clustering algorithm based upon the prognostic pyroptosis-associated gene set stratified gastric cancer specimens to different pyroptosis phenotypes, genetic changes as well as expression perturbation across above clusters remained unclear. Thus, we further developed the pyroptosis scoring system to define different pyroptosis phenotypes. We observed that pyroptosis cluster-B (immune-desert phenotype) presented the strongest pyroptosis score, followed by the cluster-A (immune-inflamed tumors) as well as the cluster-C (immune-excluded tumors). Such pyroptosis score served as a reliable prognostic biomarker for gastric cancer. Low pyroptosis score exhibited significant associations with increased somatic mutations, SCNA as well as MSI-H subtype. In our study, pyroptosis score was a preferred indicator of genome alterations. Several findings suggest that chemotherapy agents elicit tumor cell deaths through inducing pyroptosis activation [25, 26]. Pyroptosis-based chemotherapy strategy may enhance immunological effects of chemotherapeutic agents [27]. Experimental evidence suggests that activation of pyroptotic cell death can enhance cisplatin-sensitivity in gastric cancer [28]. However, the relationships between pyroptosis and sensitivity to docetaxel and paclitaxel remain unclear in gastric cancer. Due to the heterogeneity of gastric cancer individuals, the subset with increased pyroptosis score potentially responded to cisplatin/docetaxel and the subset with low pyroptosis score was more likely to benefit from paclitaxel. Thus, the performance of pyroptosis score in predicting chemotherapeutic response (cisplatin, docetaxel or paclitaxel) should be further investigated. Moreover, the pyroptosis score presented distinct correlation to predictors of the immunotherapeutic response such as TMB and IPS, suggesting that pyroptosis may affect the immunotherapeutic effects. In actual, this study confirmed the reliable predictive efficacy of the pyroptosis score in the immune response through two anti-PD-1 cohorts, suggesting that pyroptosis may be utilized for selecting the immunophenotypes as well as guide treatment regimens.

In clinical practice, the pyroptosis score can be applied for comprehensively evaluating the pyroptosis phenotypes and the corresponding TME immune cell infiltrations for individual patients, which can also infer the immune phenotypes. Moreover, it enabled to assess patients’ stages and grades, molecular subtypes, genetic mutation, etc. The pyroptosis score potentially serves as a reliable prognostic indicator for predicting clinical outcomes. Furthermore, this score showed the well performance in predicting the responses to adjuvant chemotherapy and immunotherapy. Especially, our findings yielded a few novel insights into immunotherapy for altering the pyroptosis phenotypes as well as further reshaping the adverse TME, thereby transforming “cold tumors” into “hot tumors”. Thus, our findings might provide innovative ideas for ameliorating the clinical responses to immunotherapeutic regimens, selecting distinct immune phenotypes as well as facilitating personalized immunotherapeutic regimen.

Nevertheless, several limitations should be pointed out. Firstly, there is still a lack of suitable immunotherapy-based gastric cancer cohorts. Hence, we combined two immunotherapy cohorts of metastatic melanoma cases receiving PD-1 blockade for proving the effects of the pyroptosis scoring system. Therefore, our results provided the modest evidence that pyroptosis score potentially estimates the responses to anti-PD-1 therapy for gastric cancer patients. Secondly, the pyroptosis phenotypes and pyroptosis scoring system were determined based upon retrospective datasets. Hence, prospective datasets should be applied to verify our conclusion. Thirdly, our study focuses on gastric cancer patients and their TME, and it is not clear whether our findings can be generalized to other cancer types. We will investigate the generalizability of these findings to pan-cancers in our future studies. Finally, the study only investigated the strong correlations between pyroptosis and immunomodulation in gastric cancer, but did not establish their causality. Other factors, e.g., genetic mutations or environmental factors, might contribute to both pyroptosis and immune responses. Our future studies will solve these limitations. Moreover, we investigated that pyroptosis score owned negative connections with DNA damage repair mechanisms, with positive connections with stromal-related signatures. In our future studies, we will investigate the molecular mechanisms underlying pyroptosis affecting DNA damage repair and stromal activation in the TME.

Collectively, this study comprehensively assessed the pyroptosis phenotypes across 1275 gastric cancer patients. The three pyroptosis phenotypes were characterized by distinct survival outcomes and immunophenotypes, proving pyroptosis exerts a prominent implication in the modulation of tumor immunity. Moreover, evaluation of the pyroptosis score of individual tumors might more precisely guide therapeutic strategies for individual patients.

## MATERIALS AND METHODS

### Sample curation and preprocessing

**Figure 11:**
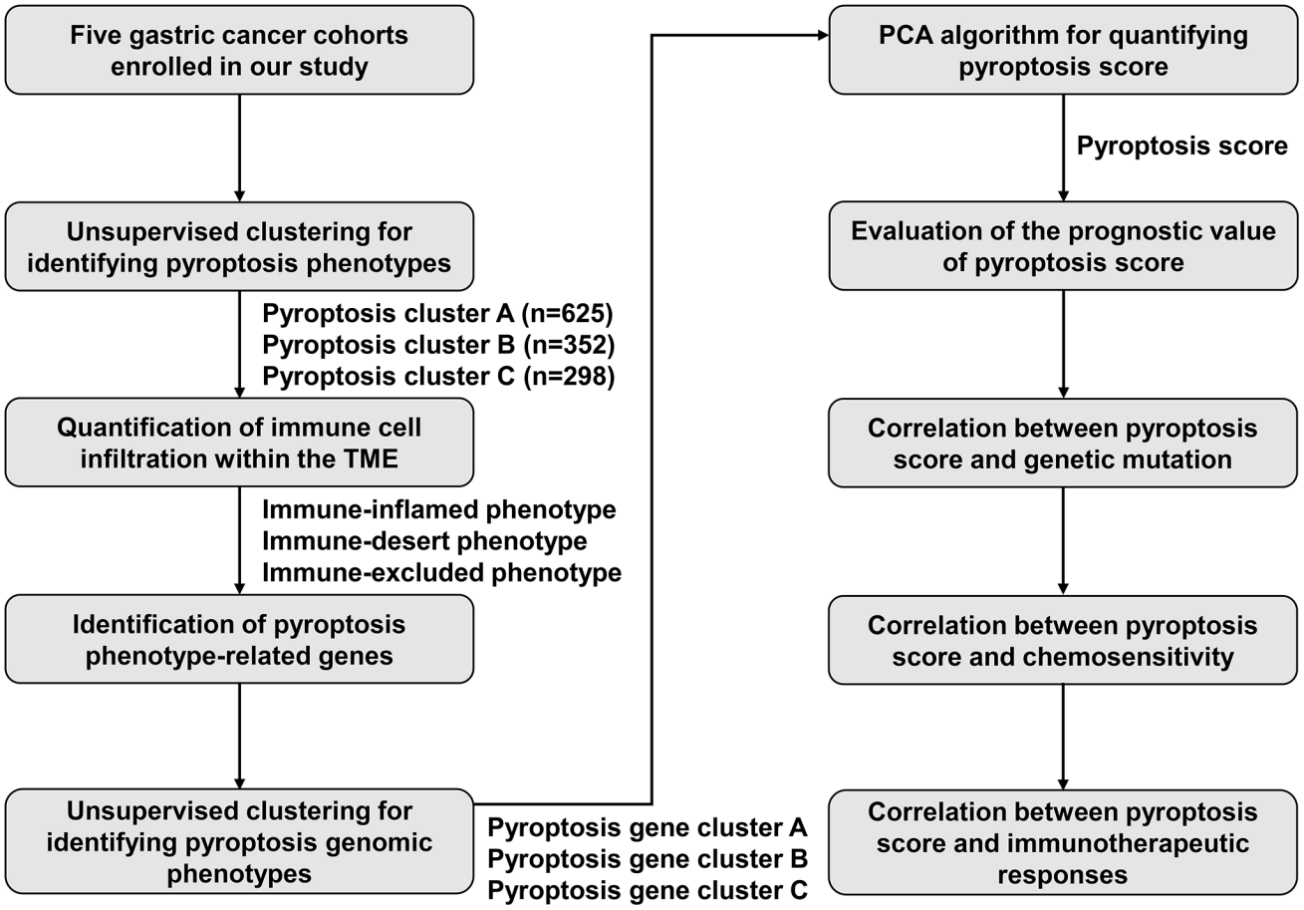
The workflow diagram in this study.

Transcriptome data or complete patient information of gastric cancer were collected from the Cancer Genome Atlas (TCGA) or Gene Expression Omnibus (GEO; https://www.ncbi.nlm.nih.gov/geo/). In total, five gastric cancer cohorts (GSE15459, GSE62254, GSE84437, GSE26253 and TCGA-stomach adenocarcinoma (STAD)) were ultimately included for further analysis. RNA-seq profiles (FPKM value) of 32 controls and 375 gastric tumors were retrieved from TCGA-STAD through the Genomic Data Commons (https://portal.gdc.cancer.gov/) with TCGAbiolinks package, with subsequent conversion of FPKM to TPM value [29]. The original “CEL” files of microarrays on the Affymetrix platform were obtained, with subsequent background adjustment and quantile standardization. Furthermore, the standardized matrix of microarrays on the Illumina platform was acquired. Utilizing “ComBat” function from sva tool, log2 (TPM + 1) was constructed [30], and the negative values that represented abnormal values were removed, and thus batch effects of integrated GSE15459, GSE62254, GSE84437 and TCGA-STAD datasets were removed. Somatic mutations or copy number variations (CNVs) were curated from TCGA. The maftools package was employed for analyzing and visualizing the somatic mutation data. Figure 11 shows the workflow diagram of this study.

### Unsupervised clustering for pyroptosis-related genes

Thirty-three pyroptosis-related genes were curated from the published studies. Their chromosomal locations were drawn via Rcircos tool [31]. Protein-protein interactions (PPIs) of 33 pyroptosis-related genes were conducted with the STRING (https://string-db.org/) [32]. Univariate cox regression analysis was applied to screen prognostic pyroptosis-related genes. By unsupervised clustering analysis, different pyroptosis phenotypes based upon prognostic pyroptosis-associated gene set were established and gastric cancer cases were classified for subsequent exploration. The amount and reliability of clustering were evaluated with consensus clustering algorithm. Through ConsensuClusterPlus tool, such steps as well as 1000 times repetitions were carried out for insuring the clustering reliability [33]. The number of clusters k was set as 2~9. Moreover, 80% of the samples were sampled utilizing a re-sampling approach. T-distributed stochastic neighbor embedding (t-SNE) was run for proving the subtype assignment utilizing above ferroptosis genes.

### Gene set variation analysis (GSVA)

The 50 hallmark gene sets were curated from the Molecular Signatures Database (MSigDB) [34]. GSVA enrichment analysis was then presented to estimate the activity of these pathways and biological processes in gastric cancer samples with GSVA package [35].

### Estimation of immune cell infiltration

Through single sample gene set enrichment analysis (ssGSEA), the relative abundance of 28 immune cells was quantified in the TME. Immune cell markers were curated from previous research [36, 37]. The relative abundance of each immune cell was normalized to ssGSEA score, ranging from 0 to 1. CIBERSORT, a deconvolution approach based upon linear support vector regression, was also applied for estimating the abundance of 22 immune cell populations [38].

### Quantifying immune response predictors

The mRNA expression levels of the main immune checkpoints (IDO1, LAG3, CTLA4, TNFRSF9, ICOS, CD80, PDCD1LG2, TIGIT, CD70, TNFSF9, ICOSLG, KIR3DL1, CD86, PDCD1, LAIR1, TNFRSF8, TNFSF15, TNFRSF14, IDO2, CD276, CD40, TNFRSF4, TNFSF14, HHLA2, CD244, CD274, HAVCR2, CD27, BTLA, LGALS9, TMIGD2, CD28, CD48, TNFRSF25, CD40LG, ADORA2A, VTCN1, CD160, CD44, TNFSF18, TNFRSF18, BTNL2, C10orf54, CD200R1, TNFSF4, CD200 and NRP1) were quantified in each gastric cancer sample. The ESTIMATE algorithm was employed for calculating immune/stromal score, thus inferring immune/stromal cell infiltration levels as well as tumor purity based upon the transcriptional profiling [39]. Tumor mutation burden (TMB) score was calculated as the amount of mutations/length of exons (30 Mb). Immunophenoscore (IPS), an indicator in predicting response of CTLA-4 or anti-PD-1 blockade, quantifies determining factors of cancer immunogenic characteristics as well as characterizes the immune profiles within the tumor and cancer antigenome [37]. IPS was calculated based on the MHC-associated signatures, checkpoint or immunomodulatory molecules, effector or suppressor cell populations.

### Identification of pyroptosis phenotype-related differentially expressed genes (DEGs)

Through limma package, DEGs (adjusted *p* < 0.05) between distinct pyroptosis phenotypes were determined [40]. Then, overlapped pyroptosis phenotype-related DEGs were extracted for further analysis.

### Construction of the pyroptosis score

The consensus clustering algorithm was adopted to define genomic clustering number and reliability according to the expression of overlapped pyroptosis phenotype-related DEGs. Prognostic analysis of each pyroptosis phenotype-related DEG was carried out utilizing univariate cox regression models. The DEGs with *p* < 0.05 were chosen for subsequent exploration. Principal component analysis (PCA) was adopted for constructing the pyroptosis score. Principal component (PC) 1 and 2 were extracted to define the pyroptosis score. Such method possessed the advantages of focusing the score on the set with the most block of well correlated (or anticorrelated) genes in the set, and down-weighting contributions from genes that don’t track with other set members. The pyroptosis score was calculated utilizing an approach like GGI [41, 42], as follows: pyroptosis score = ∑(PC1i+PC2i), of which i indicated prognostic pyroptosis phenotype-associated DEG expression.

### Functional enrichment analyses

Gene Ontology (GO) or Kyoto Encyclopedia of Genes and Genomes (KEGG) enrichment on pyroptosis phenotype-related DEGs was run with clusterProfiler package [43]. Gene set enrichment analysis (GSEA) was presented to observe the biological pathways involved in pyroptosis score based upon the “c2.cp.kegg.v6.2.symbols” set.

### Relationship of pyroptosis score with well-established biological signatures

The gene sets of several well-established biological signatures were collected according to the published literature [44–46]. Spearman correlation was carried out for assessing interactions between pyroptosis score and above biological pathways.

### Assessment of the sensitivity of chemotherapeutics

Cisplatin, docetaxel, gemcitabine, and paclitaxel were selected as candidate chemotherapeutic agents. To predict the sensitivity of these chemotherapeutic agents, IC50 of each sample was measured utilizing pRRophetic tool [47].

### Transcriptome and follow-up data of immunotherapy cohorts

Two immunotherapy cohorts were collected for our analysis: metastatic melanoma patients receiving PD-1 blockade treatment (GSE78220 cohort) [48] and Liu et al. [49]. Gene expression profiles were curated and converted to TPM values for further analysis.

### Patients and specimens

Three gastric cancer cases who had surgically proven primary gastric cancer and received D2 radical gastrectomy were recruited from Xiamen Haicang Hospital. Tumors and adjacent normal tissue specimens of three cases were collected during surgery, which were immediately placed in liquid nitrogen and stored at −80°C. This study gained the approval of the Medical Ethics Committee of Xiamen Haicang Hospital (KY-2020014). All patients provided written informed consent.

### Western blot

Radioimmunoprecipitation assay (RIPA; Beyotime, China) buffer was applied for protein extraction from tissues. The supernatants were run on 8%–12% acrylamide gels via SDS-PAGE and then transferred onto polyvinylidene difluoride membranes (Millipore, USA). Antibodies against GPX4 (#P36969; RayBiotech, USA; 1:1000), NLRP7 (#AB117732; Abcam, USA; 1:1000), CASP6 (#AB108335; Abcam; 1:1000), NLRP6 (#P59044; RayBiotech; 1:1000), IL-6 (#AB271042; Abcam; 1:2000), CASP5 (#AB40887; Abcam; 1:3000), CASP4 (#AB238124; Abcam; 1:1500), TIRAP (#P58753; RayBiotech; 1:1000), and GAPDH (#60004-1-Ig; Proteintech, China; 1:20000) and HRP-conjugated secondary antibodies (#ab97080 or ab47827; Abcam; 1:2000) were employed for western blot. The chemiluminescence western blot detection system (Bio-Rad, USA) was utilized for protein detection.

### Statistical analysis

Statistical analysis was generated by R 3.6.1. Kaplan-Meier survival analysis was run through Survminer and survival packages. Statistical significance between groups was estimated through student’s t or Wilcoxon test. Meanwhile, three groups were compared with Kruskal-Wallis test or one-way analysis of variance. The receiver operating characteristic (ROC) curve was conducted to investigate the predictive efficacy of pyroptosis score and common clinical features (age, gender, grade and stage) in gastric cancer prognosis, with subsequent estimation of area under the curve (AUC) utilizing timeROC package. *P* values were two-side, with *p* < 0.05 as statistical difference.

### Availability of data and materials

The data used to support the findings of this study are included within the supplementary information files.

## Supplementary Materials

Supplementary Figures

Supplementary Table 1

Supplementary Table 2
