# Supplementary material for: Comprehensive characterization of pyroptosis phenotypes with distinct tumor immune profiles in gastric cancer to aid immunotherapy
Source: Aging (Albany NY). 2023 Aug 17;15(16):8113–36. doi: 10.18632/aging.204958 (PMC10497016; doi:10.18632/aging.204958)
Supplement: Supplementary Figures [file aging-15-204958-s001.pdf]

SUPPLEMENTARY FIGURES

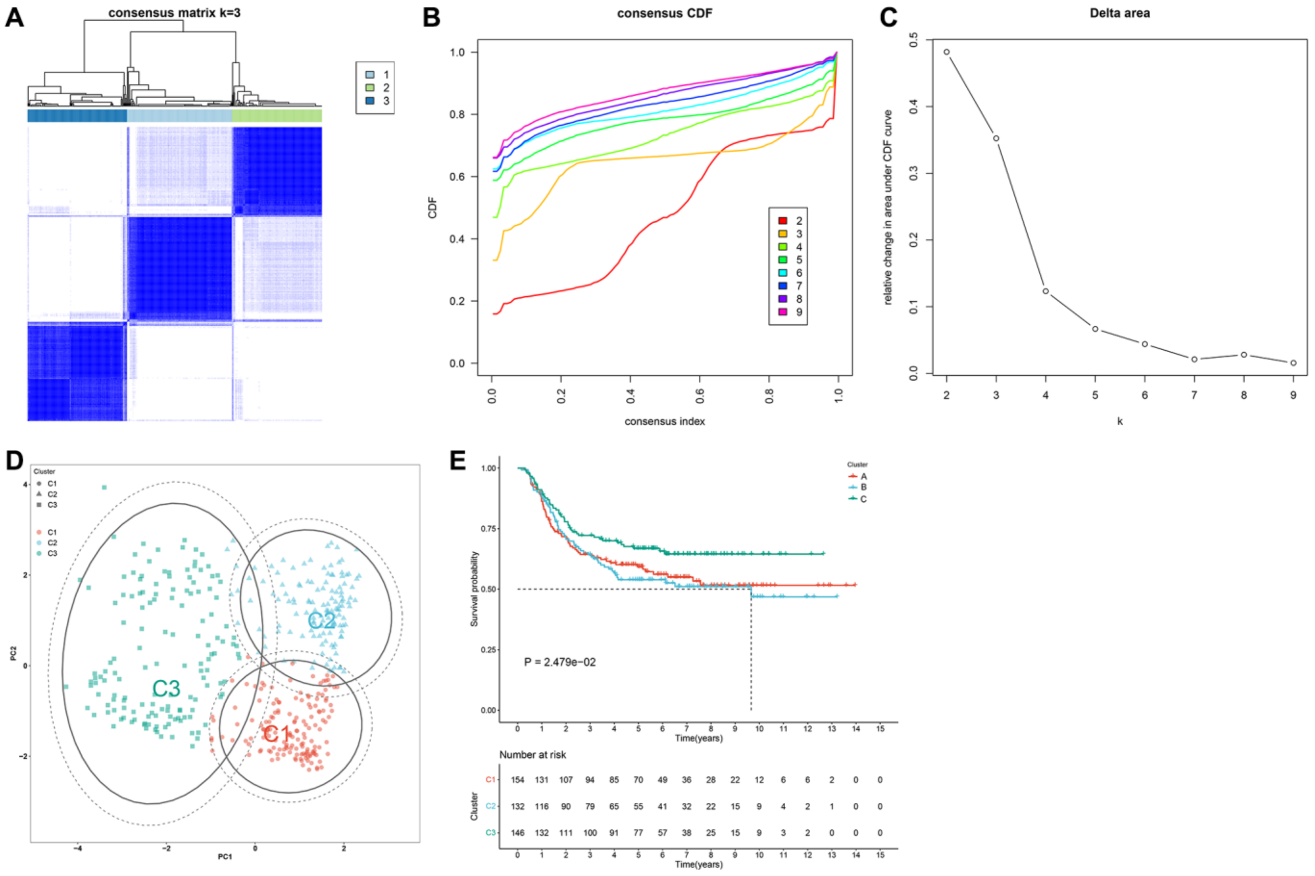

Supplementary Figure 1. External validation of pyroptosis phenotypes in the GSE26253 cohort. (**A**) Heat map for the optimal clustering by k = 3 based on prognostic pyroptosis-related genes in the GSE26253 cohort. (**B**) The consensus matrix for k = 2–9 identified by different colors. (**C**) The CDF plots of consensus clustering matrix for k = 2–9. (**D**) The t-SNE plots of the mRNA expression profiles of prognostic pyroptosis-related genes showing the three distinct subtypes indicated by different colors. (**E**) Kaplan–Meier survival curves for the three pyroptosis subtypes.

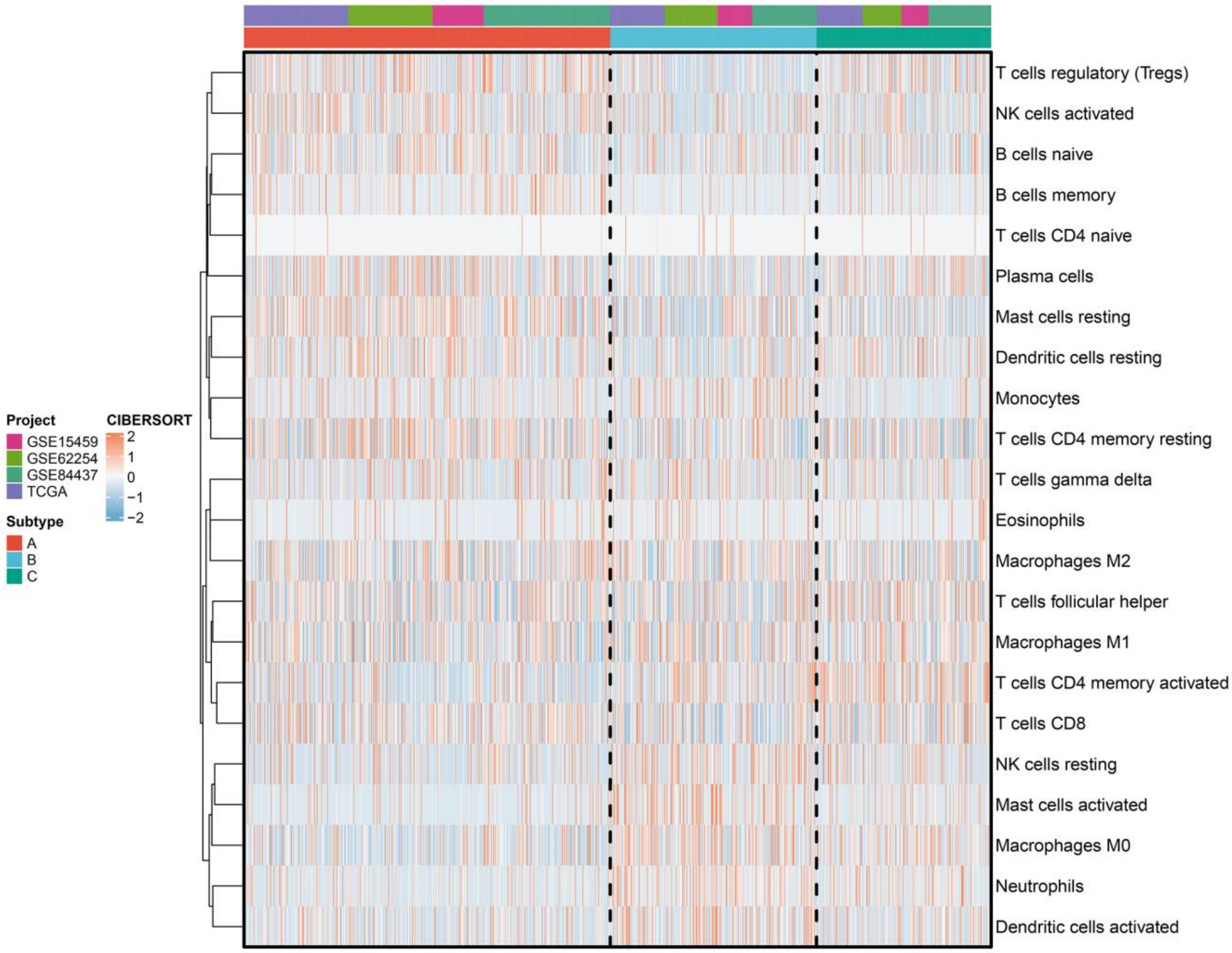

Supplementary Figure 2. Validation of immune cell infiltrations across pyroptosis phenotypes in the gastric cancer meta-cohort through CIBERSORT.
